# Dr. Ivan Pavlov

**Published:** 1916-04

**Authors:** 


**Dr. Ivan Pavlov,** the well known physiologist, recipient of the Nobel Prize in 1904, died recently at Petrograd, aged 67.

